# IL-15, IL-18 and IL-21 Along the Stress–Smoking–Periodontal Health Axis: A Cross-Sectional Study in Mexican Adults

**DOI:** 10.3390/biomedicines14010114

**Published:** 2026-01-06

**Authors:** Carmen Celina Alonso-Sánchez, Juan Manuel Guzmán-Flores, Julieta Sarai Becerra-Ruiz, Celia Guerrero-Velázquez, María Luisa Ramírez-de los Santos, Edgar Iván López-Pulido, Saúl Ramírez-de los Santos

**Affiliations:** 1 Doctorado en Biociencias, Centro Universitario de los Altos (CUALTOS), Universidad de Guadalajara, Tepatitlán de Morelos CP 47620, Jalisco, Mexico; celina.alonso@academicos.udg.mx; 2 Departamento de Clínicas, Centro Universitario de los Altos (CUALTOS), Universidad de Guadalajara, Tepatitlán de Morelos CP 47620, Jalisco, Mexico; 3 Departamento de Ciencias de la Salud, Centro Universitario de los Altos (CUALTOS), Universidad de Guadalajara, Tepatitlán de Morelos CP 47620, Jalisco, Mexico; 4 Departamento de Clínicas Odontológicas Integrales, Centro Universitario de Ciencias de la Salud (CUCS), Universidad de Guadalajara, Guadalajara CP 44340, Jalisco, Mexico; 5 Departamento de Clínicas de Salud Mental, Centro Universitario de Ciencias de la Salud (CUCS), Universidad de Guadalajara, Guadalajara CP 44340, Jalisco, Mexico; 6 Departamento de Psicología Básica, Centro Universitario de Ciencias de la Salud (CUCS), Universidad de Guadalajara, Guadalajara CP 44340, Jalisco, Mexico

**Keywords:** periodontal disease, gingivitis, IL-18, IL-21, IL-15, stress, smoking, cytokines, psychoneuroimmunology, biomarkers

## Abstract

From a psychoneuroimmunology standpoint, stress and cigarette smoking are plausible modulators of periodontal inflammation through neuroendocrine–immune pathways and cytokine networks. Interleukin-18 (IL-1 family), interleukin-21 (common γ-chain cytokine), and interleukin-15 (tissue-resident lymphocyte activation/homeostasis) are mechanistically relevant candidates to characterize in relation to these exposures. We aimed to quantify serum IL-15, IL-18, and IL-21 and examine their associations with stress, smoking, and periodontal status in Mexican adults. **Methods**: Cross-sectional study (*n* = 65; 18–60 years; 70.8% female). Smoking status (23.1% smokers) and periodontal status were recorded; due to low periodontitis frequency (*n* = 3), periodontal status was analyzed as healthy (23.1%) versus periodontal disease (76.9%; gingivitis + periodontitis). Stress was assessed using the 18-item Symptomatic Stress Questionnaire and dichotomized as no/low stress (0–10; 52.3%) versus pathological stress (11–54; 47.7%). Systolic and diastolic blood pressure were recorded. IL-15, IL-18, and IL-21 were measured in serum by immunoassay. Analyses used medians (IQR), Mann–Whitney U tests with rank-biserial effect sizes, and exploratory Benjamini–Hochberg false discovery rate (FDR) adjustment across the nine primary cytokine-by-contrast tests; correlations with age and diastolic blood pressure were exploratory. **Results**: Cytokine distributions were right-skewed, particularly for IL-21. Across smoking, stress, and periodontal-status contrasts, no comparison met q < 0.05 after FDR adjustment. Effect-size patterns were heterogeneous rather than uniformly monotonic across exposures (e.g., IL-18 showed higher central tendency in healthy vs. periodontal disease; IL-21 showed higher central tendency in no/low stress vs. pathological stress), indicating substantial inter-individual variability in circulating cytokines within this cohort. **Conclusions**: In this exploratory cross-sectional sample, serum IL-15, IL-18, and IL-21 did not show robust, multiplicity-resistant differences by smoking, stress, or periodontal status. The findings provide a transparent description of distributional properties and hypothesis-generating patterns that motivate larger, longitudinal studies with repeated cytokine sampling, standardized periodontal assessment, and improved control of key confounders to clarify the relevance of these cytokines to periodontal inflammation under behavioral exposures.

## 1. Introduction

Periodontitis is a chronic, polymicrobial, host-mediated inflammatory disease that destroys tooth-supporting tissues and may lead to tooth loss, with relevant systemic implications for public health [1,2,3,4]. Contemporary classifications and consensus statements emphasize that clinical progression reflects a dynamic interplay between dysbiotic biofilm and a dysregulated host immune response, in which cytokine networks contribute to the transition from gingival inflammation to destructive periodontal disease and to the magnitude of tissue damage [5,6,7]. At the population level, the burden of periodontal pathology remains high and heterogeneous. In Latin America and Mexico, surveillance systems report elevated prevalences of gingival inflammation and periodontal disease in young adults, supporting the need for better biological characterization of host-response profiles in these settings [8,9,10,11,12].

From a psychoneuroimmunology perspective, psychological stress and cigarette smoking may modulate periodontal inflammation through neuroendocrine–immune pathways and shifts in effector/regulatory cell function [13,14,15,16,17]. Stress has been associated with alterations in cortisol and catecholamines and with changes in pro-inflammatory and resolution-related signaling, potentially influencing bleeding, tissue repair, and susceptibility to inflammatory dysregulation [13,18,19,20]. Smoking increases systemic inflammatory burden, alters gingival microvasculature, promotes dysbiosis, and worsens periodontal treatment response while modifying local and systemic immune signatures [21,22,23,24,25]. Together, stress and smoking constitute relevant behavioral exposures that can plausibly shape inflammatory phenotypes at the gingival barrier.

Within this framework, three cytokines are mechanistically relevant candidates to examine in relation to periodontal inflammation and behavioral exposures: interleukin-18 (IL-18), interleukin-21 (IL-21), and interleukin-15 (IL-15). IL-18, an IL-1 family cytokine, promotes IFN-γ production and NF-κB-related inflammatory programs and may enhance matrix metalloproteinase expression in periodontal ligament fibroblasts, linking it to extracellular matrix remodeling [26,27,28,29]. IL-21, a common γ-chain cytokine with key roles in T cell (including Tfh/Th17-related) and B cell biology, regulates humoral immunity and chronic inflammatory signaling; elevated levels have been reported in periodontal disease in saliva and serum, consistent with a role in sustained inflammatory activation [30,31,32,33]. IL-15 supports survival and activation of tissue-resident NK and T cells and, under danger-signal conditions, can amplify cytotoxic effector circuits and chronic inflammation at barrier sites [34,35,36,37]. Taken together, these cytokines capture complementary components of innate–adaptive crosstalk that are relevant to periodontal immunobiology and to potential modulation by stress and smoking.

However, integrated evidence examining IL-18, IL-21, and IL-15 simultaneously while accounting for stress, smoking, and periodontal status remains limited, particularly in Mexican populations that are underrepresented in the international biomarker literature [8,9,10,11,12,21,22,23,24,25]. Establishing a transparent, reproducible description of these cytokines in relation to key behavioral exposures can inform the design of future longitudinal and interventional studies in which psychosocial (stress-reduction) and behavioral (smoking cessation) components are integrated with conventional periodontal care [3,13,21]. Importantly, given the cross-sectional design and moderate sample size typical of exploratory biomarker studies, inferences should be framed as hypothesis-generating rather than confirmatory.

Objective: To quantify IL-18, IL-21, and IL-15 in Mexican adults and evaluate their associations with pathological stress (Symptomatic Stress Questionnaire), smoking, periodontal status, and selected sociodemographic/clinical variables (age, sex, and blood pressure). Hypotheses: (i) IL-18 will differ by periodontal status and by smoking exposure [26,27,28,29]. (ii) IL-21 will differ between participants with pathological stress and those with no/low pathological stress and by periodontal status [30,31,32,33]. (iii) IL-15 will show smaller differences across exposure strata, consistent with a modulatory/effector role in this setting [34,35,36,37]. (iv) Exploratory associations with blood pressure will be evaluated as potential correlates of inflammatory variability.

This study provides an integrated psychoneuroimmunology-oriented description of three cytokines (IL-18, IL-21, IL-15) in relation to two major behavioral exposures (stress and smoking) and blood pressure in a cohort of Mexican adults. The findings are intended to clarify directionality and effect-size patterns to guide future adequately powered longitudinal and interventional research rather than to establish clinical utility for risk stratification at this stage [38].

## 2. Materials and Methods

We conducted an observational, analytical cross-sectional study in Mexican adults. The protocol was approved by the institutional ethics committee (CUA/CEI/DOBI001/2021). Written informed consent was obtained from all participants in accordance with the Declaration of Helsinki.

A total of 65 adults (18–60 years) were included. Sociodemographic and clinical variables comprised sex, age, current smoking status (yes/no), systolic and diastolic blood pressure (mmHg), periodontal status, and stress. Eligibility criteria were intentionally broad; potential behavioral and systemic confounders (e.g., oral hygiene practices and detailed comorbidity profiles) were not comprehensively measured and are addressed as study limitations.

Periodontal diagnosis and classification followed the 2018 AAP/EFP framework using standardized clinical recording. Examinations were conducted by a postgraduate dental resident under continuous supervision of an experienced periodontist who reviewed and confirmed all diagnoses; formal examiner calibration was not performed. Assessments were carried out in a dental chair under operatory lighting using a UNC-15 periodontal probe. Six sites per tooth were recorded (mesiobuccal, buccal, distobuccal, mesiolingual, lingual, distolingual). Probing depth (PD), clinical attachment loss (CAL), and bleeding on probing (BOP) were charted; a plaque index was recorded when available. Case definitions were operationalized as follows: gingival health (BOP < 10%, no CAL, and no pockets > 3 mm), gingivitis (BOP ≥ 10% without attachment loss), and periodontitis (interdental CAL in ≥2 non-adjacent teeth or buccal/lingual CAL ≥ 3 mm with probing depths > 3 mm). Because periodontitis cases were infrequent (*n* = 3), periodontal status was collapsed for inferential analyses into periodontal health versus periodontal disease (gingivitis + periodontitis) to avoid sparse strata.

Stress symptoms were assessed using the 18-item Symptomatic Stress Questionnaire, a self-report instrument covering psychosomatic, emotional, and cognitive stress symptoms. Each item is rated from 0 to 3, yielding a total score from 0 to 54. Based on cut-points reported in occupational samples using the Symptomatic Stress Questionnaire and related Mexican reports, scores were classified as 0–8 (normal), 9–10 (tendency), and >10 (pathological stress) [39,40]. Given sample size considerations and to reduce sparse strata and multiple comparisons, stress was analyzed as a binary exposure by combining “normal” and “tendency” versus “pathological stress,” and this operational decision was prespecified for the main contrasts.

IL-18, IL-21, and IL-15 were quantified in serum using commercial antibody-based ELISA kits: R&D Systems DuoSet^®^ Human IL-15 (DY247), R&D Systems DuoSet^®^ Human Total IL-18 (DY318) (R&D Systems, Inc., Minneapolis, MN, USA), and BioLegend ELISA MAX™ Deluxe Set Human IL-21 (433804) (BioLegend, San Diego, CA, USA). Assay dynamic ranges and limits of detection were taken from the manufacturers’ package inserts. Venous blood was obtained by standard venipuncture, processed under institutional biosafety procedures to separate serum, and stored in an ultralow-temperature freezer until analysis. Serum aliquots were thawed once only. When optical density (OD) values exceeded the upper limit of the standard curve, concentrations were estimated by extrapolation from the fitted standard curve. Absorbance was measured at 450 nm with 570 nm reference using a Multiskan™ GO microplate spectrophotometer without a cuvette port (instrument version 1.00.40; serial no. 1510-04275; manufactured by Thermo Fisher Scientific) controlled with Thermo Scientific™ SkanIt™ Software for Microplate Readers (Thermo Fisher Scientific, Waltham, MA, USA; v3.2). Standard curves were fitted. Cytokine concentrations were reported in the units specified by each manufacturer.

Continuous variables were summarized as medians (IQR) due to non-normal distributions; categorical variables were summarized as counts (%). Cytokine concentrations were compared across smoking status, stress (binary), and periodontal status (binary) using Mann–Whitney U tests. Effect sizes were reported as rank-biserial r. To address multiplicity across the nine primary cytokine-by-contrast tests (3 cytokines × 3 binary contrasts), Benjamini–Hochberg false discovery rate (FDR) adjusted q-values were reported alongside nominal *p*-values; inference was considered exploratory given the sample size. Exploratory associations of cytokines with age and diastolic blood pressure were assessed using Spearman’s rank correlation. Two-sided α = 0.05 was adopted. Visualizations used distribution plots (box/violin) with logarithmic y-axis scaling to improve readability of right-skewed cytokine values. Analyses were performed in a reproducible environment (R v4.x/Python 3.11); analysis scripts are available upon request.

A generative AI assistant (ChatGPT, OpenAI, San Francisco, CA, USA; model GPT-5.2 Thinking) was used only to support drafting and to scaffold analysis code. All analyses were executed by the authors on the original dataset, outputs were cross-checked, and all analytic decisions and interpretations were made by the authors. No AI tool was used to generate, modify, or impute data. The authors reviewed and edited all AI-assisted text/graphics and take full responsibility for the content.

## 3. Results

### 3.1. Baseline Characteristics

We analyzed 65 adults (18–60 years). Median age was 23 years (IQR 21–28). Sex distribution was female 46 (70.8%) and male 19 (29.2%). Education was primary 4 (6.2%), secondary 5 (7.7%), high school 10 (15.4%), undergraduate 37 (56.9%), and postgraduate 9 (13.8%). Current smoking was reported by 15 participants (23.1%), and 50 were non-smokers (76.9%). Periodontal status comprised healthy 15 (23.1%) and periodontal disease 50 (76.9%); within periodontal disease, gingivitis predominated (47/50), while periodontitis was infrequent (3/50). Stress was categorized as no/low stress (0–10; normal + tendency) in 34 participants (52.3%) and pathological stress (11–54) in 31 (47.7%). Systolic and diastolic blood pressure summary measures are reported in Table 1.

### 3.2. Cytokines (IL-18, IL-21, IL-15): Overall Distribution

**Figure 1:**
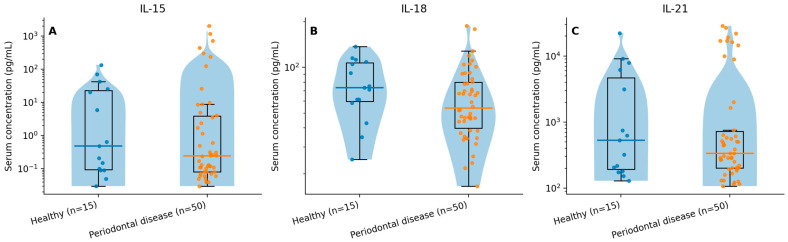
Serum cytokines by periodontal status (binary). (**A**) IL-15, (**B**) IL-18, and (**C**) IL-21 concentrations in healthy vs. periodontal disease (gingivitis + periodontitis). Violin plots display distribution density; overlaid boxplots show median and IQR; points represent individual participants. Y-axes are shown on a logarithmic scale to improve readability of right-skewed distributions.

Cytokine concentrations were right-skewed; therefore, medians (IQR) are reported in Table 2. For visualization, cytokines are displayed with a logarithmic y-axis in Figure 1 to improve readability across the observed ranges.

### 3.3. Group Comparisons by Smoking, Stress, and Periodontal Status

Pre-specified nonparametric contrasts compared IL-18, IL-21, and IL-15 across (i) smoking (yes/no), (ii) stress (pathological stress vs. no/low stress), and (iii) periodontal status (periodontal disease vs. healthy). Because periodontitis cases were few (*n* = 3), inferential analyses used a binary periodontal grouping (gingivitis + periodontitis) to avoid sparse strata. Group differences were tested using Mann–Whitney U tests, with effect sizes reported as rank-biserial r. To address multiplicity across the nine primary cytokine-by-contrast tests (3 cytokines × 3 contrasts), Benjamini–Hochberg FDR-adjusted q-values are reported alongside nominal *p*-values in Table 2. After FDR control, no comparison met q < 0.05. Directional patterns and effect sizes are therefore interpreted as exploratory.

### 3.4. Exploratory Associations with Age and Blood Pressure

Exploratory Spearman correlations of cytokines with age and diastolic blood pressure were generally small. The largest nominal association was an inverse correlation between IL-21 and age (Spearman ρ ≈ −0.27; nominal *p* ≈ 0.03), which did not remain statistically significant after FDR adjustment across these exploratory correlations. Overall, these findings are consistent with substantial biological variability in cytokine concentrations in a moderate-size, cross-sectional sample.

## 4. Discussion

This cross-sectional study profiled serum IL-18, IL-21, and IL-15 in Mexican adults and examined their associations with two behavioral exposures (smoking and stress) and periodontal status. Because periodontitis cases were rare (*n* = 3), periodontal status was analyzed as a binary grouping (healthy vs. periodontal disease). Using pre-specified nonparametric contrasts and exploratory FDR control across the nine primary tests, no comparison met q < 0.05. Accordingly, the findings should be interpreted as hypothesis-generating patterns rather than confirmatory evidence.

Mechanistically, the selected cytokines capture complementary components of periodontal immune regulation. IL-18, an IL-1 family cytokine, promotes IFN-γ-related programs and NF-κB signaling and can induce proteolytic and matrix-remodeling responses in periodontal ligament cells, providing a plausible link to connective-tissue degradation in plaque-induced inflammation [26,27,28,29]. IL-21, a common γ-chain cytokine produced by T cell subsets including Tfh/Th17-related lineages, shapes B cell help and chronic inflammatory trajectories; salivary and tissue studies have reported higher IL-21 in periodontal disease states, consistent with a role in local immune orchestration [30,31,32,33]. IL-15 supports survival and activation of tissue-resident NK and T cells and can function as a danger-signal amplifier at barrier sites, plausibly contributing to chronic inflammatory persistence under sustained exposure conditions [34,35,36,37]. These pathways align with contemporary models in which periodontal disease reflects dysregulated host–microbe homeostasis rather than a purely infection-driven process [1,4,5,6].

In this dataset, the directionality of several group differences did not follow a uniform “higher-with-worse-exposure” pattern across cytokines and strata. For example, IL-21 showed substantial dispersion and a right-skewed distribution, with higher central tendency in the no/low stress group than in the stress group, and IL-18 showed higher central tendency in healthy participants than in the periodontal disease group. Given the cross-sectional design, moderate sample size, and biological variability in circulating cytokines, such patterns may reflect heterogeneous immunophenotypes, timing/state effects, and residual confounding (e.g., oral hygiene behaviors, recent inflammatory exposures, medication use) that were not comprehensively measured. Therefore, the most defensible interpretation is that serum IL-18/IL-21/IL-15 profiles in this cohort are variable and do not provide robust, multiplicity-resistant evidence of monotonic inflammatory escalation across stress, smoking, or periodontal disease categories.

From a psychoneuroimmunology perspective, the null results after FDR adjustment remain informative. Stress and smoking have mechanistic links to periodontal inflammation via neuroendocrine–immune modulation and altered barrier-site immunity, and prior work supports associations of these exposures with inflammatory burden and impaired periodontal healing trajectories [13,14,15,16,17,21,22,23,24,25]. However, detecting small effects in circulating cytokines likely requires larger samples, repeated measures to address within-person variability, and tighter control of exposure intensity and local periodontal ecology. In particular, single time-point serum cytokines may be insensitive to localized periodontal inflammatory activity compared with site-specific matrices (e.g., gingival crevicular fluid), and stress is a subjective construct that can be influenced by unmeasured psychosocial factors and contextual stressors.

A translational context also supports cautious interpretation of systemic biochemical markers in oral health research. Interventional evidence suggests that periodontal therapy may modulate oxidative stress biomarkers and glycemic control in diabetic populations, illustrating how biochemical indicators can change alongside periodontal management in specific clinical contexts [41]. Reviews also emphasize that systemic metabolic regulation can influence local tissue responses and clinical outcomes in implant therapy, reinforcing an oral–systemic framework in which systemic status may shape interpretation of oral inflammatory findings [42]. These lines of evidence motivate integrated biomarker approaches, but they do not establish clinical utility for any single cytokine marker in the present exploratory dataset.

Clinical implications: The current findings do not support clinical risk stratification or monitoring based on IL-18/IL-21/IL-15 in this sample. The main contribution is descriptive: documenting the distributional properties and exploratory between-group patterns of these cytokines in Mexican adults while transparently addressing multiplicity and sparse disease strata. Research implications: Future work should prioritize (i) adequately powered longitudinal or interventional designs with repeated cytokine sampling to quantify within-person variability and temporal coupling with periodontal status; (ii) standardized assessment of oral hygiene and periodontal inflammation severity to reduce residual confounding; and (iii) integrated exposure phenotyping, including smoking intensity/dose and stress characterization, to test mechanistic pathways more directly. Where feasible, combining systemic measurements with site-specific periodontal matrices and microbiome or oxidative-stress measures may improve sensitivity to local disease biology.

Strengths and limitations: Strengths include biologically coherent cytokine selection, pre-specified nonparametric analyses with effect-size reporting, exploratory multiplicity control, and parsimonious presentation. Limitations include cross-sectional design (no causal inference), moderate sample size, single time-point cytokine measurement (these values should be considered semi-quantitative and interpreted with caution), rare periodontitis cases requiring binary periodontal grouping, broad eligibility criteria with limited measurement of key confounders (including oral hygiene), and the stress assessment.

In summary, serum IL-18, IL-21, and IL-15 showed substantial heterogeneity in this cohort, and no between-group contrast remained significant after FDR adjustment. These findings support a cautious, hypothesis-generating interpretation and motivate larger, better-controlled longitudinal studies to clarify whether these cytokines capture meaningful host-response variation related to stress, smoking, and periodontal inflammation.

## 5. Conclusions

In this cross-sectional study of Mexican adults, serum IL-18, IL-21, and IL-15 displayed substantial inter-individual variability and right-skewed distributions. Using pre-specified nonparametric comparisons and exploratory FDR control across the primary tests, no between-group contrast (by smoking, pathological stress, or periodontal status) met q < 0.05. Therefore, the observed effect-size patterns should be interpreted as hypothesis-generating rather than confirmatory. The cytokine panel remains biologically relevant given contemporary models of dysregulated host–microbe homeostasis in periodontal disease and the established roles of IL-18 (IL-1 family), IL-21 (γc-chain/Tfh–Th17-related biology), and IL-15 (tissue-resident lymphocyte activation) in barrier-site inflammation [1,4,5,6,26,27,28,29,30,31,32,33,34,35,36,37].

Exploratory correlations with age and diastolic blood pressure were generally small and did not provide stable evidence after multiplicity adjustment. Clinically, the present data do not support biomarker-based risk stratification or monitoring using IL-18/IL-21/IL-15 in this sample. Future research should prioritize adequately powered longitudinal or interventional designs with repeated cytokine sampling, standardized periodontal assessment and plaque control, and improved measurement of key confounders (including oral hygiene and exposure intensity) to test whether these cytokines add prognostic or mechanistic value beyond established clinical indices and population burden [8,9,10,11,12].

## Figures and Tables

**Table 1:** Sociodemographic and clinical characteristics of the sample (*n* = 65).

Variable	Value
Age (years), median (IQR)	23 (21–28)
SBP (mmHg), median (IQR)	108 (100–120)
DBP (mmHg), median (IQR)	76 (70–80)
Female	46 (70.8%)
Male	19 (29.2%)
Primary	4 (6.2%)
Secondary	5 (7.7%)
High school	10 (15.4%)
Undergraduate	37 (56.9%)
Postgraduate	9 (13.8%)
Non-smoker	50 (76.9%)
Smoker	15 (23.1%)
Healthy	15 (23.1%)
Periodontal disease	50 (76.9%)
No/low stress	34 (52.3%)
Pathological stress	31 (47.7%)

Data are shown as median (IQR) or *n* (%), as appropriate. Periodontal disease includes gingivitis (*n* = 47) and periodontitis (*n* = 3). Three-category classification (normal/tendency/pathological). Abbreviations: SBP, systolic blood pressure; DBP, diastolic blood pressure; IQR, interquartile range; Symptomatic Stress Questionnaire (18 items).

**Table 2:** Serum IL-15, IL-18, and IL-21 by smoking, stress and periodontal status (*n* = 65).

Cytokine (pg/mL)	Non-Smoker (*n* = 50)	Smoker (*n* = 15)	*p* (q)	No/Low Stress (*n* = 34)	Pathological Stress (*n* = 31)	*p* (q)	Healthy (*n* = 15)	Periodontal Disease (*n* = 50)	*p* (q)
IL-15	0.40 (0.08–9.57)	0.13 (0.11–0.24)	0.188 (0.564)	0.21 (0.10–5.72)	0.26 (0.08–6.67)	0.963 (0.963)	0.48 (0.10–23.01)	0.24 (0.08–3.91)	0.570 (0.944)
IL-18	60.43 (43.39–89.39)	65.56 (46.25–86.06)	0.827 (0.944)	63.59 (43.94–83.33)	52.40 (44.22–91.58)	0.839 (0.944)	73.67 (59.92–107.05)	53.93 (39.95–79.77)	0.109 (0.564)
IL-21	328.6 (202.5–5444.8)	483.9 (179.9–556.5)	0.591 (0.944)	526.2 (207.0–8650.8)	289.8 (199.1–561.0)	0.145 (0.564)	530.8 (193.8–4676.5)	337.0 (202.5–728.3)	0.720 (0.944)

Data are median (IQR). Group comparisons used Mann–Whitney U tests (two-sided). q-values are Benjamini–Hochberg FDR-adjusted across the nine primary tests (3 cytokines × 3 binary contrasts). No/low stress = 0–10; pathological stress = 11–54. Periodontal disease = gingivitis + periodontitis.

## Data Availability

The data presented in this study are available upon reasonable request from the corresponding author. The datasets are not publicly available due to privacy considerations and restrictions imposed by the institutional ethics committee (protocol code CUA/CEI/DOBI001/2021). De-identified analysis datasets, the data dictionary, and analysis notes can be shared upon request and subject to a data-use agreement.
